# New Insights into the Low-Temperature Properties of the Ternary Halide Na_2_CrCl_4_: Magnetic Ordering and Entropy Determination

**DOI:** 10.1021/acs.jpcc.5c08600

**Published:** 2026-04-15

**Authors:** Nick T. H. ter Veer, Ian M. Berkel, Indu Dhiman, Jean-Christophe Griveau, Eric Colineau, Andries van Hattem, Sebastian Drange Couweleers, Rudy J. M. Konings, Anna L. Smith

**Affiliations:** † Radiation Science & Technology Department, Faculty of Applied Sciences, 2860 Delft University of Technology, Mekelweg 15, Delft 2629 JB, The Netherlands; ‡ 145265 European Commission, Joint Research Centre, Karlsruhe D-76125, Germany

## Abstract

The structural, thermodynamic, and magnetic properties of Na_2_CrCl_4_ have been investigated to provide fundamental insights into this ternary halide relevant to chloride-based molten salt reactor systems. Room-temperature powder X-ray and neutron diffraction confirm a monoclinic (*P*2_1_/*c*) structure and phase purity. Neutron diffraction measurements at 4.6 K reveal additional magnetic reflections indexed with $k=\left(\frac{1}{2},0,0\right)$ , indicating the onset of long-range antiferromagnetic order. Low-temperature heat capacity measurements in the range 2–300 K show a pronounced λ-type anomaly at *T*_N_ = 8.5 ± 0.5 K, with an associated magnetic entropy *S*_mag_ = 11.9 ± 0.4 J K^–1^ mol ^–1^ consistent with antiferromagnetic ordering of high-spin Cr^2+^ (*S* = 2), a second-order phase transition. The standard molar entropy at 298.15 K, *S*_m_^°^(298.15 K) = 256.8 ± 7.7 J K^–1^ mol ^–1^, is slightly lower than previous CALPHAD assessments of the NaCl-CrCl_2_ system. Magnetic susceptibility measurements also confirm antiferromagnetic behavior, with a Curie–Weiss fit giving μ_eff_ = 5.57 ± 0.05 μ_B_ and θ_CW_ = −15.0 ± 1.0 K. Compared to the related ferromagnetic chlorides K_2_CrCl_4_, Rb_2_CrCl_4_, and Cs_2_CrCl_4_, Na_2_CrCl_4_ exhibits a distinctly lower ordering temperature and antiferromagnetic structure, likely due to variations in lattice geometry and exchange interactions. These results provide the first experimental thermodynamic parameters for Na_2_CrCl_4_, contributing to refining phase diagrams and corrosion models in chloride salt systems.

## Graphical abstract

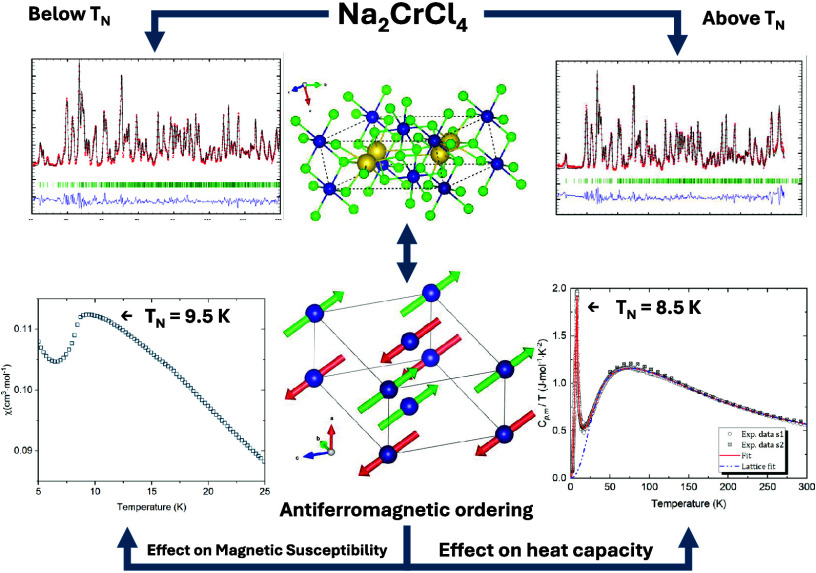

## Introduction

Molten Salt Reactors (MSRs) are an advanced class of nuclear fission systems in which the primary coolant and, in most designs, the nuclear fuel itself exist in a molten salt state. These reactors offer several advantages over conventional solid-fuel reactors, including improved thermal efficiency, passive safety mechanisms, and the ability to operate at high temperatures and near atmospheric pressure.^1^ Historically, most research has focused on fluoride-based fuel salts due to their chemical stability and established use in early MSR experiments.^2^ However, chloride-based MSRs have gained increasing attention in recent years, particularly for their suitability in fast-spectrum reactor designs, because their lower neutron moderation enables efficient breeding and transmutation of nuclear fuels.^3^ This renewed interest in chloride salts presents opportunities for further innovation but also introduces new challenges, particularly concerning corrosion and the long-term behavior of structural materials-molten salt interactions.

Structural materials in MSRs must withstand the highly corrosive environments of the fuel salts. Various nickel-based alloys with chromium, iron, and molybdenum as alloying elements to enhance corrosion resistance, were developed and extensively tested in the molten salt reactor experiment (MSRE).^4^ These tests revealed that chromium is preferentially depleted from the alloy, thereafter dissolved in the fuel salt, and subsequently redeposited in cooler regions of the reactor system. In chloride salts, a similar phenomenon is expected and has been observed, where studies and operation of microloops with chloride salts revealed the significant depletion of chromium.^5–7^ As chromium depletes from structural alloys, it reacts with the chloride-based fuel salt, leading to the dissolution of chromium chloride corrosion products into the fuel salt mixture. The most common base salt for chloride-MSR systems is NaCl, within a fuel salt mixture of typical compositions NaCl-KCl-UCl_3_, NaCl-MgCl_2_–PuCl_3_, NaCl-ThCl_4_–PuCl_3_.^8–10^ A recent investigation by Tiwari et al. into the NaCl-CrCl_2_ system, relevant for understanding into the effect of CrCl_2_ on the base fuel phase equilibria, reveals that only a single stable intermediate compound, Na_2_CrCl_4_, forms under equilibrium conditions.^11^

Understanding the thermochemical and thermophysical properties of Na_2_CrCl_4_ is essential for evaluating fuel-salt behavior throughout reactor operation. These properties influence the tendency of Na_2_CrCl_4_ to precipitate in colder parts of the core and primary circuit and are also important for predicting salt behavior during solidification events. Previous studies on structurally analogous compounds, such as X_2_CrCl_4_ with X = (K, Rb, Cs), have revealed ferromagnetic ordering at low temperatures,^12–14^ suggesting that similar behavior may occur in Na_2_CrCl_4_, making it a particularly intriguing phase. Such magnetic phase transitions can significantly impact thermodynamic properties, including heat capacity and entropy, which are crucial for modeling the stability and evolution of salts in MSR environments.

In this article, neutron diffraction at 4.6 K, low-temperature heat capacity and magnetic susceptibility measurements on Na_2_CrCl_4_ are reported, in the temperature ranges 2 to 300 K and 5 to 300 K, respectively, providing insight into the thermodynamics and magnetic properties of this ternary halide compound. Together, these techniques provide a consistent picture of the compound’s magnetic and thermodynamic behavior. Our study reveals an antiferromagnetic transition that manifests in the heat capacity, affecting the standard entropy at 298.15 K.

## Experimental Section

### Synthesis

Disodium tetrachlorochromate­(II) (Na_2_CrCl_4_) was synthesized via a solid-state reaction using high-purity sodium chloride (NaCl) and chromium­(II) dichloride (CrCl_2_). All samples were handled inside an Ar-filled glovebox with low oxygen and water content (<5 ppm) and not exposed to air at any stage of the experiments. The starting materials, NaCl and CrCl_2_ (both from Merck, 99.999% metals basis), were mixed in a stoichiometric 2:1 molar ratio. The mixture was thoroughly ground in an agate mortar to ensure homogeneity.

Following grinding, the powder was transferred to a vacuum-sealed borosilicate ampule to prevent oxidation. The sealed ampule was heated at 700 K for 150 h, allowing for the formation of Na_2_CrCl_4_.

### Diffraction Analysis

#### X-ray Diffraction (XRD)

The formation and purity of Na_2_CrCl_4_ were confirmed by powder X-ray Diffraction (XRD). Measurements were performed using a PANalytical X′Pert PRO diffractometer equipped with a Cu–K_α_ radiation source. The instrument was operated at 45 kV and 40 mA in Bragg–Brentano geometry.

Data were collected over a 2θ range of 10° to 120° with a step size of 0.008° and a total acquisition time of approximately 11 h. To prevent oxidation and moisture contamination, the sample was prepared and loaded into an airtight sample holder inside an argon-filled glovebox. The holder was sealed with Kapton foil to maintain an inert environment during measurement.

Structural analysis was conducted using the profile refinement method developed by Loopstra, van Laar and Rietveld within the FullProf Suite.^15–17^ The refinement process confirmed phase purity, and no secondary phases were detected within the instrument’s resolution. The purity of the prepared material is estimated >99%.

#### Neutron Diffraction (ND)

Neutron diffraction (ND) measurements were conducted at the PEARL beamline of the Hoger Onderwijs Reactor at Delft University of Technology.^18^ To ensure sample integrity and prevent interaction with atmospheric moisture or oxygen, the material was encapsulated in a vanadium null-alloy container inside the glovebox, which was hermetically sealed using a rubber O-ring.

Data were collected at room temperature, as well as at 4.6, 15, and 100 K, using a fixed neutron wavelength of 1.66718 Å. The diffraction patterns were recorded over a 2θ range of 11° to 159°. Structural refinement and phase analysis were carried out using the FullProf suite, employing the profile refinement method to extract lattice parameters and atomic positions while also resolving the magnetic structure.

### Heat Capacity Measurements

The low-temperature heat capacity was measured using two complementary thermal-relaxation calorimetric setups: a Quantum Design Physical Property Measurement System (QD-PPMS) in the temperature range of 2–300 K, and a Quantum Design Versalab system for measurements between 50 and 290 K. Both systems employ the thermal relaxation method, in which a known heat pulse is applied and the resulting temperature relaxation of the sample is monitored to determine the heat capacity.

Samples were pressed into pellets and encapsulated in Stycast 2850FT^19^ under inert conditions to prevent degradation from air or moisture. Details of the masses of each measured sample are presented in the Supporting Information. The encapsulated pellets were subsequently mounted onto the calorimeter puck using Apiezon N grease to ensure good thermal contact. The contribution of the grease N and sample puck was independently measured (addenda curve) and together with the contribution of Stycast subtracted (calibration equation by Javorsky et al.^20^) from the total heat capacity signal.

Measurements were performed in both a zero magnetic field and in an applied field of 7, 9, and 14 T to investigate field-dependent effects. No correction for impurity phases was required, as phase purity of the samples was confirmed by X-ray diffraction. A 3% uncertainty on the measured heat capacity was used in the error analysis.

A pronounced anomaly in the heat capacity was observed near the magnetic ordering temperature, consistent with an antiferromagnetic transition. To isolate the magnetic contribution to the heat capacity, the lattice background was modeled using a combined Debye–Einstein approach. The heat capacity due to lattice vibrations, *C*_lat_(*T*), was fitted to the experimental data outside the magnetic transition region using the expression

1 $$C_{\mathrm{lat}}\left(T\right)=n_{D}\cdot D\left(\theta_{D},T\right)+n_{E}\cdot E\left(\theta_{E},T\right)$$

where *n*_D_ + *n*_E_ ≈ *N*_atoms_ in the formula unit. The Debye and Einstein contributions are given by

2 $$D\left(\theta_{D},T\right)=9R{\left(\frac{T}{\theta_{D}}\right)}^{3}\int_{0}^{\Theta_{D}/T}\frac{x^{4}e^{x}}{{\left(e^{x}-1\right)}^{2}}dx$$

3 $$E\left(\theta_{E},T\right)=3R{\left(\frac{\theta_{E}}{T}\right)}^{2}\frac{e^{\theta_{E}/T}}{{\left(e^{\theta_{E}/T}-1\right)}^{2}}$$

where *R* is the gas constant, and θ_D_, θ_E_ are the Debye and Einstein temperatures, respectively. In the lowest temperature range (below 5 K), the lattice contribution was moreover fitted to a harmonic lattice model

4 $$C_{\mathrm{lat}}\left(T\right)=\Sigma_{n}B_{n}T^{n},n=3,5,7,9,\cdot \cdot \cdot$$

The λ anomaly itself was fitted with a combination of polynomial functions. The magnetic heat capacity was then extracted as

5 $$C_{\mathrm{mag}}\left(T\right)=C_{p}^{\exp}\left(T\right)-C_{\mathrm{lat}}\left(T\right)$$

and the magnetic entropy was obtained by integrating

6 $$S_{\mathrm{mag}}\left(T\right)=\int_{0}^{298.15K}\frac{C_{\mathrm{mag}}\left(T\right)}{T}dT$$

This analysis allows quantification of the entropy change associated with the antiferromagnetic ordering in Na_2_CrCl_4_.

### Magnetic Susceptibility Measurements

Low-temperature magnetic susceptibility measurements were carried out using a Quantum Design MPMS-XL SQUID magnetometer. Approximately 1.5 mg of finely ground sample powder was loaded into a nonmagnetic plastic straw under an argon atmosphere and sealed to prevent degradation.

DC magnetic susceptibility was measured over the temperature range of 5–300 K under an applied magnetic field of 1 T. Measurements were performed during heating, with a temperature step size of 0.2 K. No distinction was made between zero-field-cooled (ZFC) and field-cooled (FC) protocols. Given the small mass and geometry, demagnetisation effects were considered negligible.

The raw magnetization data (in emu) was converted to molar magnetic susceptibility, χ_mol_, in units of cm^3^ mol^–1^ using the sample mass and the molar mass of Na_2_CrCl_4_. No explicit correction was applied for core diamagnetism or for the sample holder background. The susceptibility data were analyzed using a Curie–Weiss fit in the paramagnetic region to extract the effective magnetic moment and Weiss temperature.

## Results and Discussion

### Structural Characterization

The phase purity and crystal structure of Na_2_CrCl_4_ were first examined by room-temperature powder X-ray diffraction (XRD). The measured diffraction pattern (Figure 1) could be indexed with a monoclinic unit cell (*P*2_1_/*c*) and was successfully fitted using a profile refinement method. The extracted lattice parameters are summarized in Table 1, and in good agreement with those of Kanno et al.^21^ and Lutz et al.^22^ No secondary phases were detected within the detection limits of the measurement.

**Table 1:** Refined Structural Parameters of Na_2_CrCl_4_ (*P*2_1_/*c*, 14) in This Work at Different Temperatures Using ND and XRD Compared to Structural Data Reported by Kanno et al.^21^ and Lutz et al.^22^ RT = Room Temperature

*a* (Å)	*b* (Å)	*c* (Å)	β (°)	temp. (K)	ref
3.9407(2)	11.5942(7)	6.9632(4)	92.444(2)	RT	this work [XRD]
3.9407(1)	11.5905(5)	6.9618(3)	92.465(2)	RT	Kanno et al. [XRD][Bibr ref21]
3.9416(2)	11.590(2)	6.965(2)	92.48(1)	RT	Lutz et al. [XRD][Bibr ref22]
3.939(6)	11.586(3)	6.959(8)	92.44(1)	295	This work [ND]
3.913(9)	11.526(7)	6.915(8)	92.14(6)	100	this work [ND]
3.907(6)	11.518(6)	6.906(2)	92.06(5)	15	this work [ND]
3.906(2)	11.519(8)	6.906(3)	92.05(8)	4.6	this work [ND]

**Figure 1:**
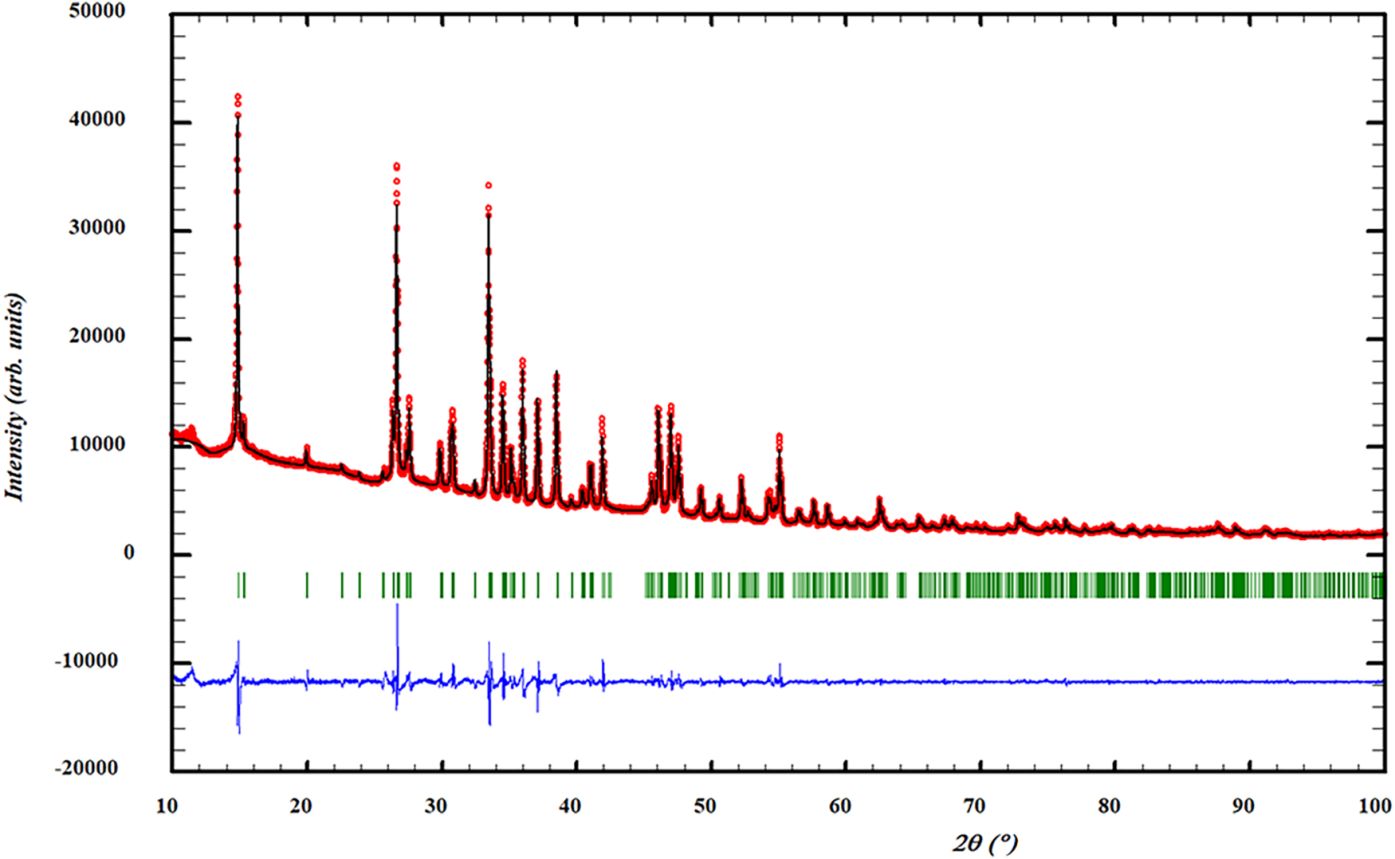
Profile refinement of X-ray diffraction data of Na_2_CrCl_4_. The depicted graph shows the observed intensity represented by the red line (*Y*_obs_) juxtaposed with the calculated intensity from the refinement (*Y*_calc_, depicted by the black line). The difference between the two is visually demonstrated by the blue line (*Y*_obs_ – *Y*_calc_). Furthermore, the vertical lines denote the positions of Bragg reflections. The measurement was performed at λ = Cu–K_α_.

The crystal structure of Na_2_CrCl_4_ consists of isolated CrCl_6_ octahedra, in which each Cr^2+^ ion is coordinated by six chloride ions in a slightly distorted octahedral geometry as seen in Figure 2. Sodium ions are surrounded by six to eight chloride ions, forming irregular coordination polyhedra, and are located between the CrCl_6_ octahedra. The CrCl_6_ units are arranged in edge-sharing chains along the crystallographic *b*-axis. This one-dimensional connectivity may be relevant for the anisotropic magnetic interactions leading to antiferromagnetic ordering discussed later. Unlike the structurally related compounds Rb_2_CrCl_4_ ^23^ and Cs_2_CrCl_4_,^14^ which adopt orthorhombic and tetragonal structures respectively and exhibit ferromagnetic ground states, Na_2_CrCl_4_ displays a markedly different structure and magnetic behavior. The geometry of the superexchange paths between Cr^2+^ ions mediated via Cl^–^ anions plays a critical role in determining the sign and strength of magnetic interactions. In Na_2_CrCl_4_, the Cr–Cl–Cr angles are close to 180°, favoring antiferromagnetic coupling according to the Goodenough–Kanamori–Anderson (GKA) rules.^24,25^ In contrast, the heavier alkali analogues exhibit more 90 °Cr–Cl–Cr angles, which tend to favor ferromagnetic exchange due to the orbital orthogonality.

**Figure 2:**
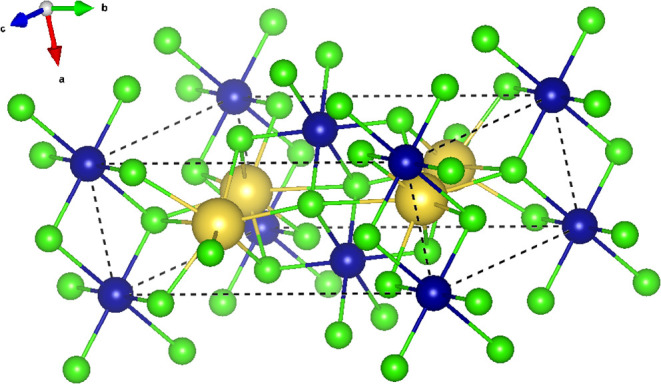
Crystal structure of Na_2_CrCl_4_ at room temperature, visualized using VESTA.^26^ The structure adopts a monoclinic symmetry (*P*2_1_/*c*). Chlorine atoms are shown in green, sodium in yellow, and chromium in blue. We propose that antiferromagnetic ordering of Cr, octahedrally coordinated by Cl, emerges as the unit cell contracts below a critical size.

Neutron diffraction (ND) measurements performed at room temperature (Figure 3) confirmed the cell parameters derived from XRD. Profile refinement of the ND data yielded lattice parameters in good agreement with those obtained from XRD (Table 1), and the refined atomic positions are listed in Table 2.

**Table 2:** Refined Atomic Positions of Na_2_CrCl_4_ as Measured by X-ray Diffraction (XRD) at Room Temperature and Neutron Diffraction (ND) at Temperatures 4.6, 15, 100, and 295 K

temperature (K)	label	*X*/*a*	*Y*/*b*	*Z*/*c*	Wyckoff
RT (XRD)	Cl1	0.0096(5)	0.1960(4)	0.1228(5)	4e
	Na	0.5163(25)	0.1800(8)	0.4064(13)	4e
	Cl2	0.5578(7)	0.4512(3)	0.2526(4)	4e
	Cr	0	0	0	2a
295 (ND)	Cl1	0.0115(8)	0.1959(4)	0.1157(5)	4e
	Na	0.5082(25)	0.1809(8)	0.4114(13)	4e
	Cl2	0.5580(7)	0.4518(3)	0.2590(4)	4e
	Cr	0	0	0	2a
100 (ND)	Cl1	0.0094(6)	0.1966(3)	0.1162(4)	4e
	Na	0.5099(19)	0.1804(6)	0.4087(10)	4e
	Cl2	0.5556(6)	0.4521(3)	0.2580(4)	4e
	Cr	0	0	0	2a
15 (ND)	Cl1	0.0094(7)	0.1964(3)	0.1163(4)	4e
	Na	0.5099(20)	0.1803(6)	0.4093(10)	4e
	Cl2	0.5550(7)	0.4522(3)	0.2574(4)	4e
	Cr	0	0	0	2a
4.6 (ND)	Cl1	0.0085(86)	0.1964(3)	0.1164(5)	4e
	Na	0.5072(25)	0.1800(8)	0.4086(13)	4e
	Cl2	0.5558(8)	0.4520(3)	0.2571(5)	4e
	Cr	0	0	0	2a

**Figure 3:**
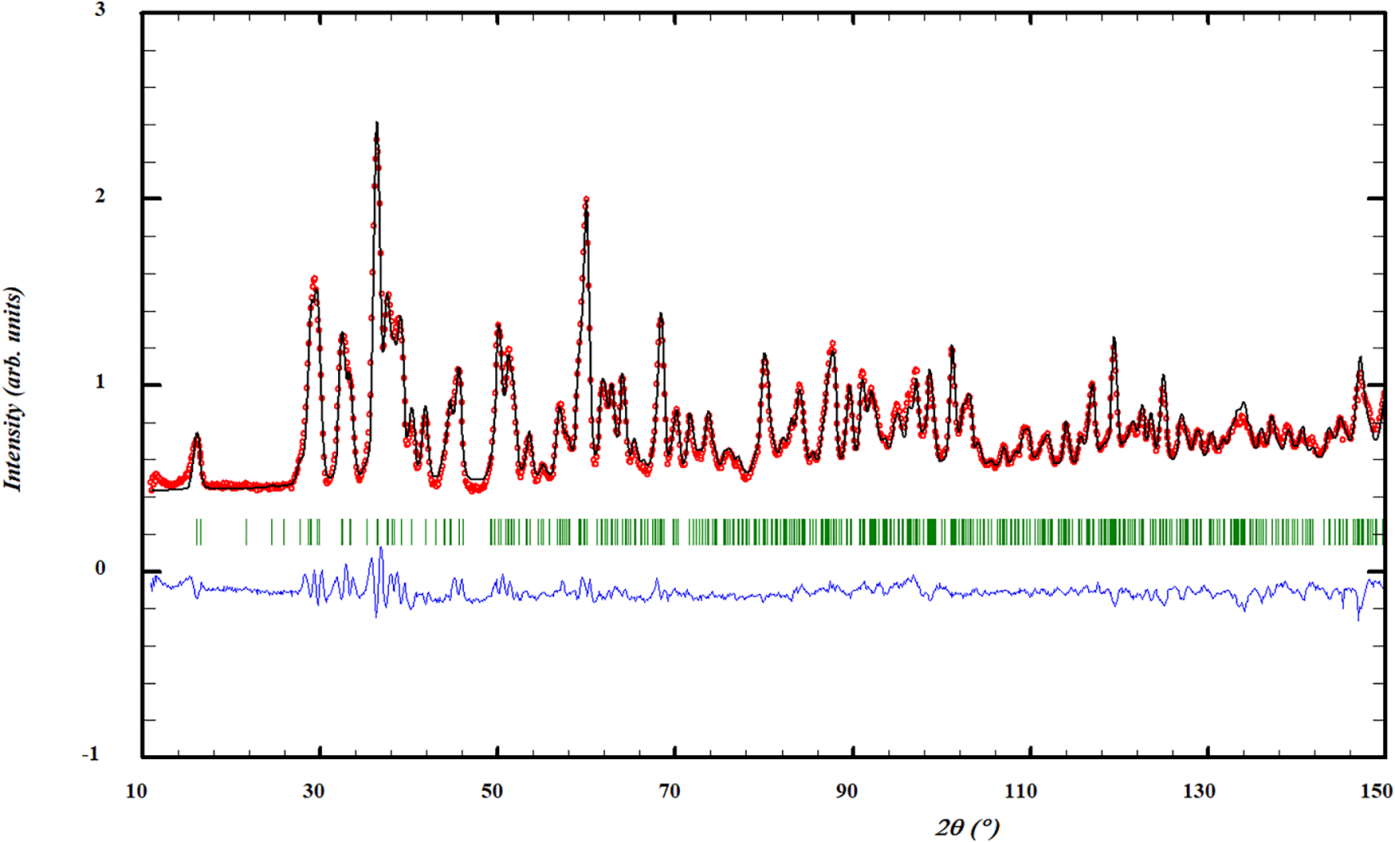
Profile refinement of neutron diffraction data at 295 K of Na_2_CrCl_4_. The depicted graph shows the observed intensity represented by the red line (*Y*_obs_) juxtaposed with the calculated intensity from the refinement (*Y*_calc_, depicted by the black line). The difference between the two is visually demonstrated by the blue line (*Y*_obs_ – *Y*_calc_). Furthermore, the vertical lines denote the positions of Bragg reflections. The measurement was performed at λ = 1.66718 Å.

At low temperature (4.6 K), additional magnetic reflections appeared in the ND pattern, indicative of long-range antiferromagnetic order. This structure corresponds to a magnetic propagation vector $k=\left(\frac{1}{2},0,0\right)$ , indicating an antiferromagnetic arrangement of Cr^2+^ moments oriented predominantly within the *a*–*c* plane. In the absence of any detectable crystallographic symmetry lowering, the magnetic symmetry can be described by a Shubnikov group derived from *P*2_1_/*c*. At the point-group level, this corresponds to the antiferromagnetic class 2/m1′, consistent with a centrosymmetric collinear antiferromagnetic state. A magnetic structure model consistent with this propagation vector was refined against the 4.6 K data (Figure 4), yielding an ordered moment of approximately 2.5 μ_B_ per Cr site. This magnetic structure is visualized in Figure 5. We note that, given the use of powder neutron diffraction data, the magnetic point group cannot be determined uniquely, but the proposed symmetry is the simplest one compatible with the refined magnetic structure. Given that the ND data collected at 15 K, shows no magnetic reflections, we can conclude that the Néel temperature, *T*_N_, is constrained between 4.6 and 15 K. No structural phase transition or symmetry lowering of the nuclear lattice was detected down to 4.6 K, aside from thermal contraction (see Supporting Information for data at 100 and 15 K).

**Figure 4:**
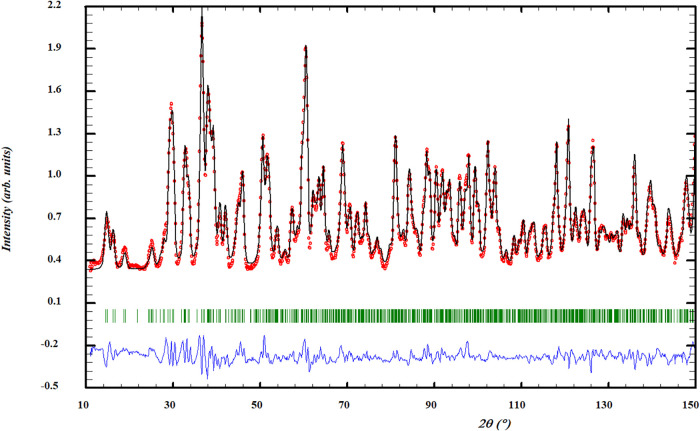
Profile refinement of neutron diffraction data at 4.6 K of Na_2_CrCl_4_. The depicted graph shows the observed intensity represented by the red line (*Y*_obs_) juxtaposed with the calculated intensity from the refinement (*Y*_calc_, depicted by the black line). The difference between the two is visually demonstrated by the blue line (*Y*_obs_ – *Y*_calc_). Furthermore, the vertical lines denote the positions of Bragg reflections. The measurement was performed at λ = 1.66718 Å.

**Figure 5:**
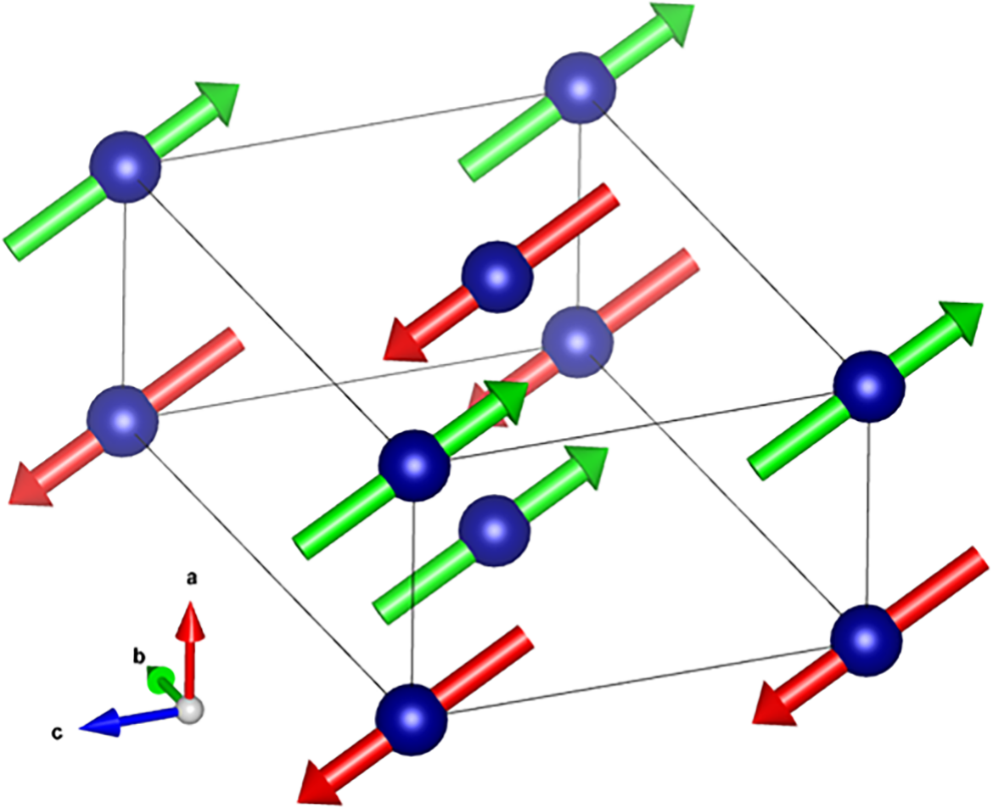
Magnetic structure of Na_2_CrCl_4_ at 4.6 K, visualized using VESTA.^26^ Blue spheres represent Cr atoms, while red and green arrows denote the orientation of the ordered magnetic moments. The structure corresponds to the magnetic propagation vector $k=\left(\frac{1}{2},0,0\right)$ , indicating an antiferromagnetic arrangement of Cr moments oriented predominantly within the *a*–*c* plane.

### Low-Temperature Heat Capacity of Na_2_CrCl_4_

The heat capacity of Na_2_CrCl_4_ was measured in the temperature range 1.9–300 K using two complementary setups, as described earlier. The evolution at zero magnetic field as a function of temperature is shown in Figure 6. A pronounced λ-type anomaly is observed at *T*_N_ = 8.5 ± 0.5 K, indicative of long-range magnetic ordering. The sharp, continuous λ-shaped divergence of *C*_p_(*T*), without any detectable discontinuity or latent heat, is characteristic of a continuous (second-order) phase transition associated with the onset of antiferromagnetic order. This value of *T*_N_ is in good agreement with the transition boundaries determined from neutron diffraction measurements, which place *T*_N_ between 4.6 and 15 K.

**Figure 6:**
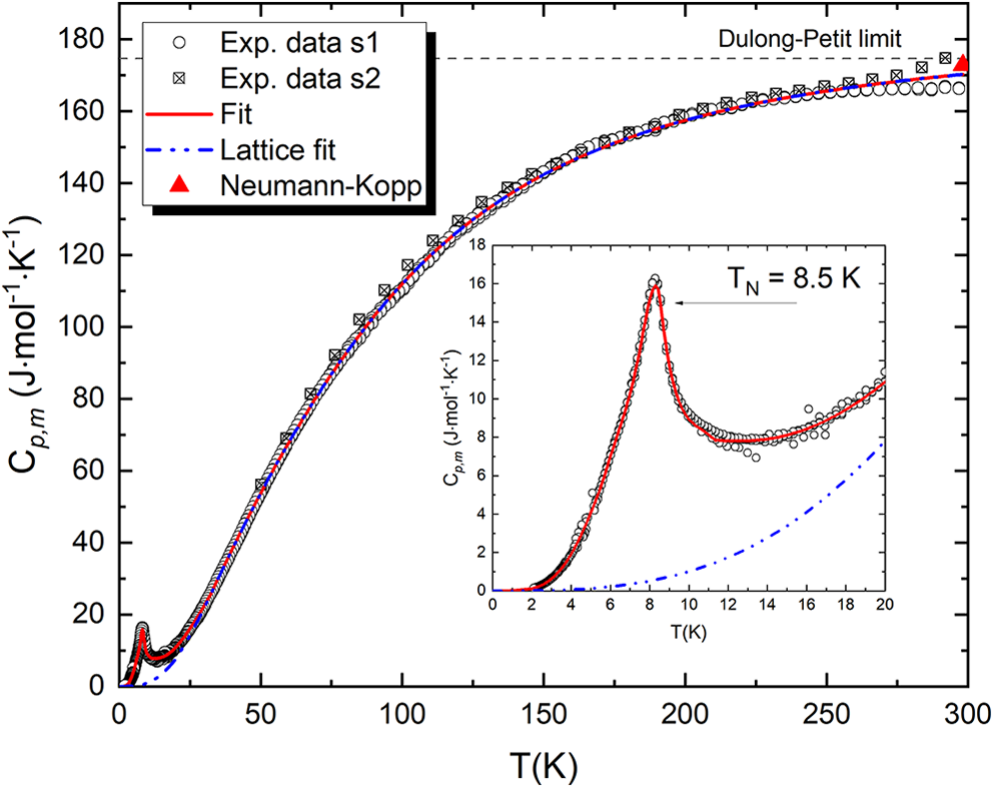
Experimentally measured heat capacity of Na_2_CrCl_4_ in the temperature range 1.9–300 K. Sample 1 (s1): open circles, Sample 2 (s2): cross squares using two complementary setups, PPMS and VersaLab, respectively. The blue dashed line corresponds to the fitted lattice contribution, obtained from a Debye–Einstein model and harmonic lattice model. The solid red line represents the total fit including both phonon and magnetic contributions.

To further investigate the magnetic transition, heat capacity measurements were performed with applied magnetic fields of 7, 9, and 14 T as shown in Figure 7. With increasing field strength, the magnitude of the λ-type anomaly progressively reduced, shifted to lower temperatures, and the peak broadened, consistent with the suppression of antiferromagnetic correlations by the external field. This behavior confirms the antiferromagnetic magnetic origin of the transition at *T*_N_.

**Figure 7:**
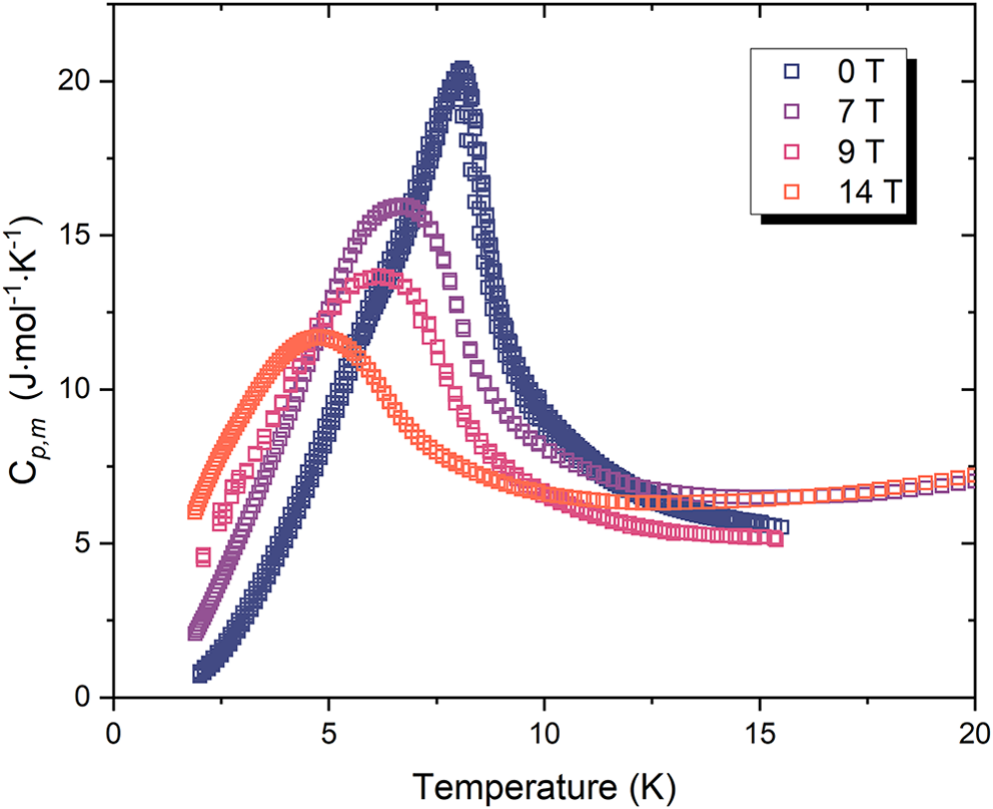
Molar heat capacity *C*_p,m_ of Na_2_CrCl_4_ measured in applied magnetic fields of 0, 7, 9, and 14 T. The sharp λ-type anomaly observed at 0 T corresponds to the magnetic ordering transition. Increasing magnetic field progressively suppresses and broadens this peak, consistent with field-induced suppression of antiferromagnetic order.

The lattice contribution to the heat capacity was modeled using a combined Debye–Einstein approach in the temperature range 30 to 298.15 K and a harmonic lattice model below 5 K. The fit yielded a Debye temperature θ_D_ = 198.3 K and an Einstein temperature θ_E_ = 372.9 K, with a respective contribution of *n*_D_ = 4.03 mol and *n*_E_ = 3.27 mol. Together, these sum to 7.30, slightly above the expected value of 7 for Na_2_CrCl_4_, which reflects the limitations of this simplified vibrational model.

Figure 8 shows *C*_p,m_/*T* as a function of temperature, along with the Debye–Einstein lattice fit (blue curve) and combined numerical fits used for interpolation across the magnetic anomaly. The magnetic contribution to the entropy *S*_mag_, was extracted by subtracting the phonon background from the measured heat capacity curve yielding 11.9 ± 0.4 J K^–1^ mol^–1^, closely approaching the expected value of *R *ln­(5) = 13.38 J K^–1^ mol^–1^ for a spin *S* = 2 system. This confirms that the observed transition is associated with the ordering of high-spin Cr^2+^ ions. The heat capacity at 298.15 K derived from the fit of the data is 170.2 ± 5.2 J K^–1^ mol^–1^. The standard molar entropy (*S*_m_^°^), was determined to be 256.8 ± 7.7 J K^–1^ mol^–1^ from integration of *C*_p,m_/*T*. To our knowledge, this is the first direct experimental determination of standard molar entropy for Na_2_CrCl_4_. Previously, only CALPHAD assessments reported values for this quantity: Tiwari et al.^11^ estimated *S*_m_^°^ = 261.59 J K^–1^ mol^–1^, while Yingling et al.^27^ reported *S*_m_^°^ = 266.7 J K^–1^ mol^–1^. Our experimentally derived entropy is thus slightly lower than these literature values but remains consistent within the expected uncertainties [Table 3].

**Figure 8:**
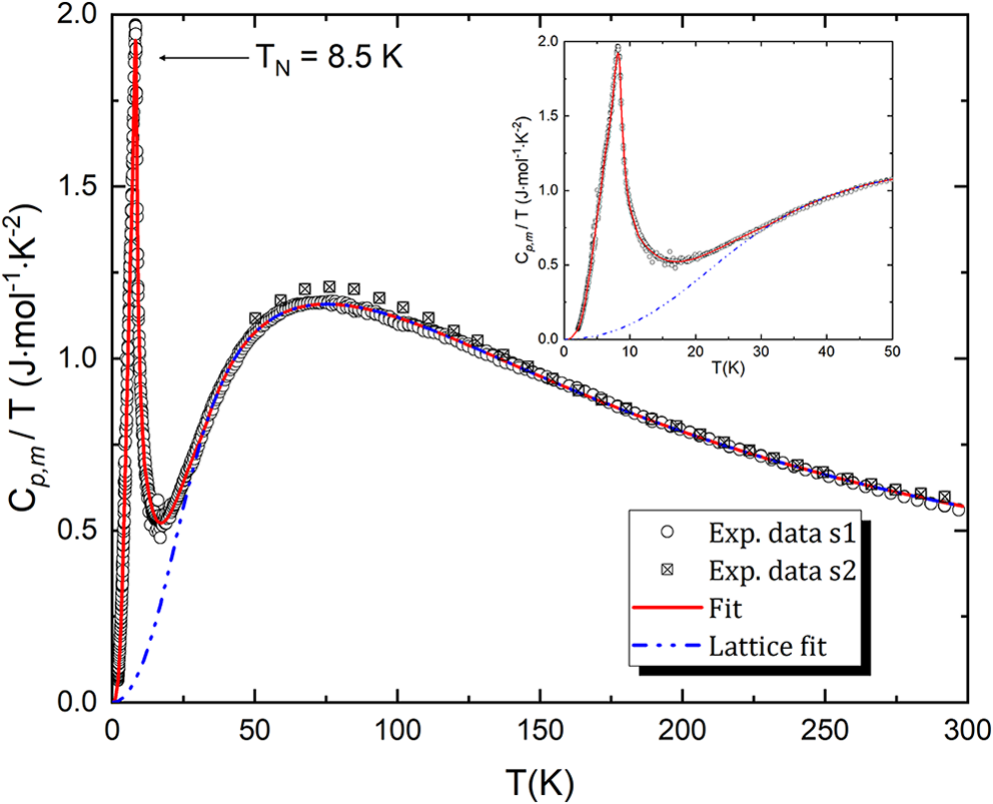
Experimentally measured *C*_p,m_/T of Na_2_CrCl_4_ in the temperature range 1.9–300 K. Sample 1 (s1): open circles, Sample 2 (s2): cross squares using two complementary setups, PPMS and VersaLab, respectively. The blue dashed line corresponds to the fitted lattice contribution, obtained from a Debye–Einstein model. The solid red line represents the total fit including both phonon and magnetic contributions.

**Table 3:** Standard Molar Entropy (*S*_m_^°^), of Na_2_CrCl_4_ from This Work and CALPHAD Assessments by Tiwari et al.^11^ and Yingling et al.^27^

source	method	*S*_m_^°^ J K^–1^ mol^–1^
this work	experimental	256.8 ± 7.7
Tiwari et al.^11^	CALPHAD assessment	261.59
Yingling et al.^27^	CALPHAD assessment	266.7

### Magnetic Susceptibility

The magnetic susceptibility, χ­(*T*), of Na_2_CrCl_4_ was measured between 5 and 300 K in an applied magnetic field of 1 T on a powder sample (Figure 9). At low temperature, χ­(*T*) exhibits a clear cusp at *T*_N_ = 9.5 ± 0.5 K, characteristic of long-range antiferromagnetic ordering. This Néel temperature is slightly higher than the value obtained from heat capacity measurements (*T*_N_ = 8.5 K), a common observation for antiferromagnetic systems due to differences in the thermodynamic and magnetic response functions.

**Figure 9:**
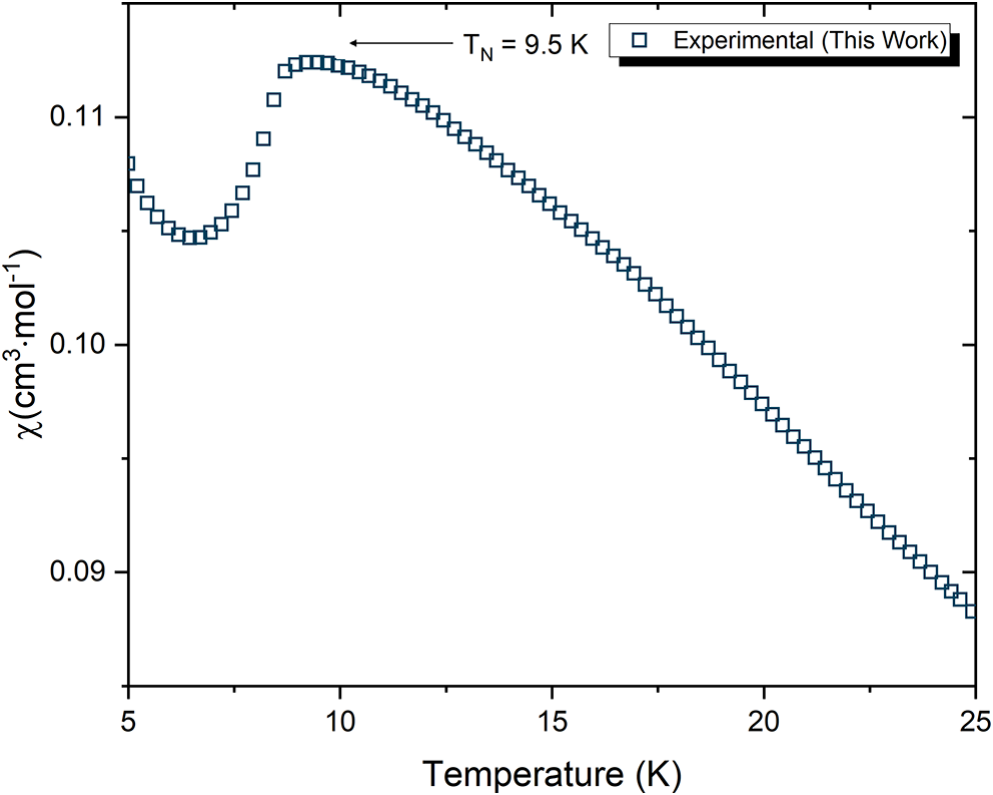
Molar magnetic susceptibility, χ­(*T*), of Na_2_CrCl_4_ measured under an applied magnetic field of 1 T. A clear cusp is observed at *T*_N_ = 9.5 K, indicating the onset of long-range antiferromagnetic ordering. The susceptibility decreases below *T*_N_.

To quantify the paramagnetic behavior above *T*_N_, the inverse molar susceptibility, χ^–1^(*T*), was fitted to the Curie–Weiss law in the temperature range 30–300 K (Figure 10), corresponding to approximately three times *T*_N_. The fit yields a Curie constant *C* = 4.00 cm^3^ K mol^–1^ and a Weiss temperature θ_CW_ = −15 K, consistent with predominant antiferromagnetic interactions. The effective magnetic moment, calculated from *C*, is μ_eff_ = 5.57 ± 0.05 μ_B_ per Cr^2+^ ion, slightly higher than the spin-only value for high-spin Cr^2+^ (μ_eff,spin_ = 4.90 μ_B_), indicating a small contribution from orbital angular momentum.

**Figure 10:**
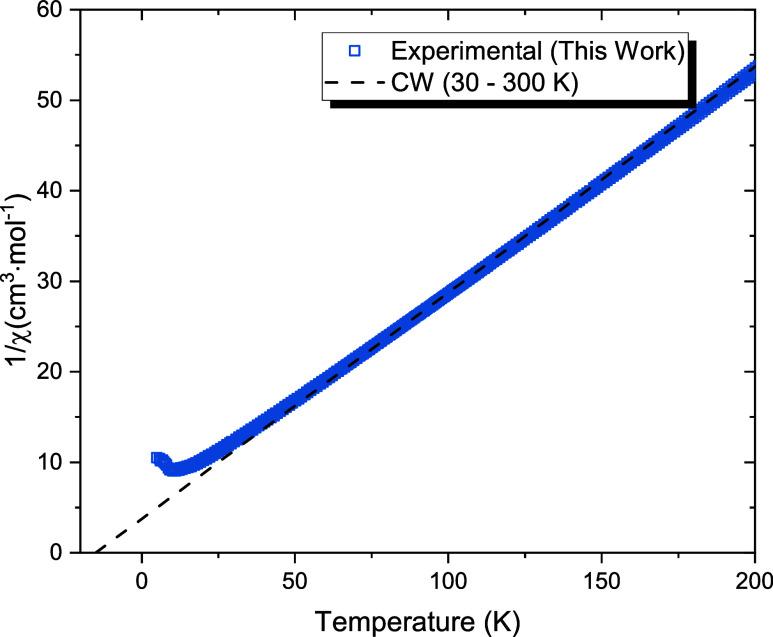
Inverse molar magnetic susceptibility, χ^–1^(*T*), of Na_2_CrCl_4_ measured in an applied field of 1 T. The dashed line represents a Curie–Weiss fit performed in the temperature range 30–300 K, yielding an effective magnetic moment μ_eff_ = 5.57 ± 0.05 μ_B_ and a Weiss temperature θ_CW_ = −15.0 ± 1.0 K.

When compared to analogous Cr^2+^ chloride compounds from literature, the extracted μ_eff_ of Na_2_CrCl_4_ is in excellent agreement. Reported values for μ_eff_ K_2_CrCl_4_ (5.34–5.61 μ_B_),^28,29^ Rb_2_CrCl_4_ (5.80 μ_B_),^28,29^ and Cs_2_CrCl_4_ (5.53–5.78 μ_B_ ^28,29^ ) demonstrate similar magnitudes, confirming the assignment of high-spin Cr^2+^ in Na_2_CrCl_4_ [Table 4].

**Table 4:** μ_eff_ (μ_B_/Cr^2+^) of Na_2_CrCl_4_ and Related Cr^2+^ Chlorides

compound	μ_eff_ (μ_B_/Cr^2+^)	ref
Na_2_CrCl_4_	5.57 ± 0.05	this work
K_2_CrCl_4_	5.34	Larkworthy^28^
K_2_CrCl_4_	5.54	Leech et al.^29^
K_2_CrCl_4_	5.61	Leech et al.^29^
Rb_2_CrCl_4_	5.80	Larkworthy^28^
Rb_2_CrCl_4_	5.80	Gregson et al.^29^
Cs_2_CrCl_4_	5.78	Larkworthy^28^
Cs_2_CrCl_4_	5.53	Leech et al.^29^

## Conclusion

This study establishes a comprehensive investigation into the structural, thermodynamic, and magnetic properties of Na_2_CrCl_4_, a ternary halide relevant to chloride-based molten salt reactor systems. The combined X-ray and neutron diffraction data confirm that the compound crystallizes in a monoclinic (*P*2_1_/*c*) structure built from isolated CrCl_6_ octahedra, where the geometry of the superexchange pathways governs the observed antiferromagnetic exchange. The onset of long-range magnetic order below 10 K and the recovery of nearly the full magnetic entropy expected for high-spin Cr^2+^ indicate that the magnetic moments are well localized.

Beyond the magnetic behavior, these measurements yield the first experimental determination of the standard molar entropy and low-temperature heat capacity of Na_2_CrCl_4_. These can be directly implemented into CALPHAD assessments of the NaCl–CrCl_2_ system, thereby reducing reliance on estimated or interpolated values.

More broadly, the identification of an antiferromagnetic ground state, in contrast to the ferromagnetism of heavier alkali analogues, highlights the sensitivity of exchange interactions to lattice geometry and cation size, an insight that may extend to other mixed-halide or mixed-alkali systems.

From an applied standpoint, the obtained thermodynamic quantities improve the understanding of chromium chemistry during salt freezing in molten-salt reactor environments, particularly in relation to corrosion phenomena that arise when NaCl containing molten salt interacts with chromium containing structural materials.

Future work should focus on re-evaluating the NaCl–CrCl_2_ system within the CALPHAD framework using the experimentally determined entropy reported here. Given the relevance of Na_2_CrCl_4_ as a key intermediate phase, additional thermodynamic measurements, such as high-temperature calorimetry and determination of the standard enthalpy of formation through solution calorimetry, are encouraged to further enhance the thermodynamic understanding of this compound. Such data will enable a more complete and accurate thermodynamic description for reactor-relevant system modeling.

## Supplementary Material



## Notes

Heat capacity sample masses and Stycast encapsulation weights (Table S1); neutron diffraction profile refinements at 100 and 15 K (Figures S1–S2) (PDF)
