# Supplementary material for: New Insights into the Low-Temperature Properties of the Ternary Halide Na2CrCl4: Magnetic Ordering and Entropy Determination
Source: J Phys Chem C Nanomater Interfaces. 2026 Apr 15;130(17):6304–12. doi: 10.1021/acs.jpcc.5c08600 (PMC13137256; doi:10.1021/acs.jpcc.5c08600)
Supplement: Supplementary file 1 [file jp5c08600_si_001.pdf]

# Supporting Information: New insights into the low temperature properties of the ternary halide $Na_2CrCl_4$: magnetic ordering and entropy determination

N. T. H. ter Veer,[†] I. M. Berkel,[†] I. Dhiman,[†] J. -C. Griveau,[‡] E. Colineau,[‡] A. van Hattem,[†] S. Couweleers,[†] R. J. M. Konings,[†] and A. L. Smith[*,†]

†*Radiation Science & Technology Department, Faculty of Applied Sciences, Delft University of Technology, Mekelweg 15, Delft, 2629 JB, The Netherlands*

‡*European Commission, Joint Research Centre, Karlsruhe, Germany*

E-mail: a.l.smith@tudelft.nl

## Supporting Information Available

Table S1: Overview of all $Na_2CrCl_4$ samples measured for heat capacity, showing the mass of $Na_2CrCl_4$ and the corresponding Stycast encapsulation weight for each sample.

| Sample # | Weight $Na_2CrCl_4$ (mg) | Weight Stycast (mg) | Total Weight (mg) |
|---|---|---|---|
| 1 | 15.19 | 1.40 | 16.59 |
| 2 | 22.71 | 2.39 | 25.10 |

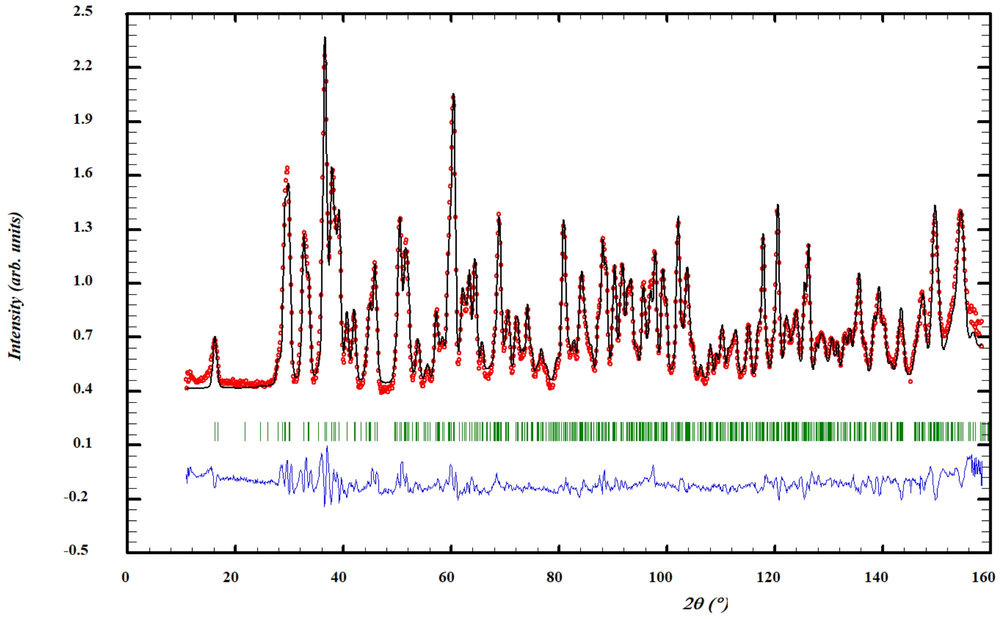


Figure S1: Profile refinement of neutron diffraction data at 100 K of $Na_2CrCl_4$. The depicted graph shows the observed intensity represented by the red line ($Y_{obs}$) juxtaposed with the calculated intensity from the refinement ($Y_{calc}$, depicted by the black line). The difference between the two is visually demonstrated by the blue line ($Y_{obs}$-$Y_{calc}$). Furthermore the vertical lines denote the positions of Bragg reflections. It is noted that the measurement was performed at $\lambda = 1.66718$ Å

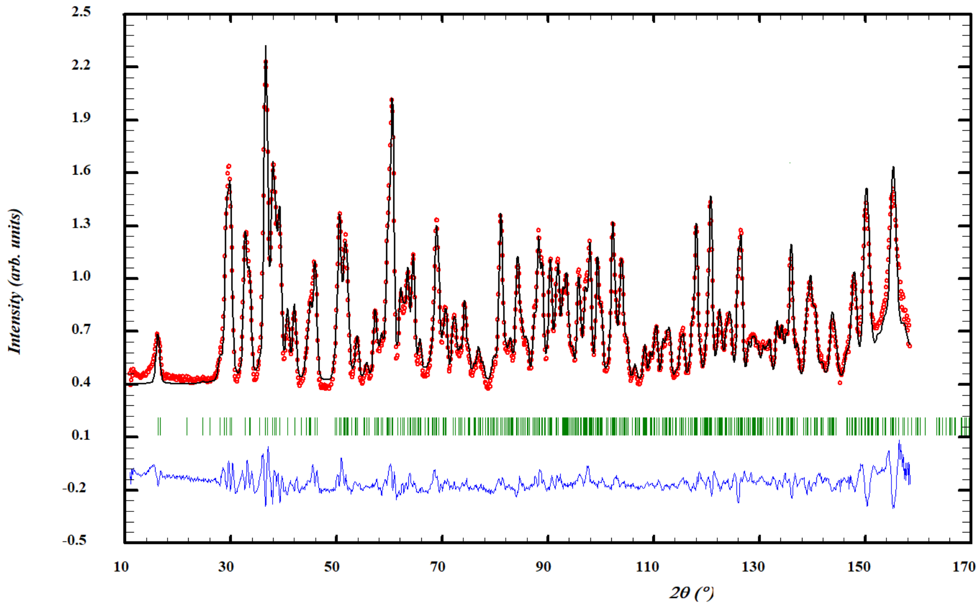


Figure S2: Profile refinement of neutron diffraction data at 15 K of $Na_2CrCl_4$. The depicted graph shows the observed intensity represented by the red line ($Y_{obs}$) juxtaposed with the calculated intensity from the refinement ($Y_{calc}$, depicted by the black line). The difference between the two is visually demonstrated by the blue line ($Y_{obs}$-$Y_{calc}$). Furthermore the vertical lines denote the positions of Bragg reflections. It is noted that the measurement was performed at $\lambda = 1.66718$ Å
